# The complete mitochondrial genome of the sea urchin, *Echinometra* sp. EZ

**DOI:** 10.1080/23802359.2018.1532335

**Published:** 2018-11-14

**Authors:** Remi N. Ketchum, Melissa B. DeBiasse, Joseph F. Ryan, John A. Burt, Adam M. Reitzel

**Affiliations:** a Department of Biological Sciences, University of North Carolina at Charlotte, Charlotte, NC, USA;; b Whitney Laboratory for Marine Bioscience, University of Florida, St. Augustine, FL, USA;; c Center for Genomics and Systems Biology, New York University Abu Dhabi, Abu Dhabi, UAE

**Keywords:** *Echinometra*, species complex, Persian/Arabian Gulf, sea urchin, mitochondrial genome

## Abstract

The complete mitogenome of *Echinometra* sp. EZ has been described and fully annotated in this study. Phylogenetic analysis of cytochrome c oxidase subunit I (COI) from six *Echinometra* species confirms that our sample is *E*. sp. EZ. The mitogenome is 15,698 bp in length and contains 13 protein-coding genes, 22 tRNAs, 2 rRNAs, and a non-coding region with an identical organization to other Echinoidea. The *E.* sp. EZ mitogenome shared ∼99.1% identity to the published *Echinometra mathaei* mitogenome, differing by 147 SNPs. The *E.* sp. EZ mitogenome will serve as a resource that can be applied to disentangling the *Echinometra* species complex and to future population genetic studies of this ecologically important sea urchin species.

## Introduction

Sea urchins belonging to the genus *Echinometra* have been widely studied and recognized for their unique patterns of genetic structure and speciation (Mcartney et al. 2000; Lessios 2006; Bronstein and Loya 2013). *Echinometra* is a pantropical genus with a distribution across the Indo-Pacific, Caribbean, and Atlantic, and plays an important role in coral reef ecosystems as a major bioeroder (Moulin et al. 2015). *Echinometra* species present in the Indo-Pacific region are best understood as a complex consisting of four distinct species. *Echinometra mathaei* and *E. oblonga* correspond to types ‘B’ and ‘D,’ respectively (Arakaki and Uehara 1999; Mita et al. 2004). The other two species are referred to as *E*. sp. A and *E.* sp. C (Palumbi et al. 1997). A recent study suggests a potential new species or subspecies that occurs in the Gulf of Aqaba/Eilat and reefs surrounding Zanzibar, referred to as *E.* sp. EZ (Bronstein and Loya 2013). Genetic and comparative genomic studies on this species complex will be valuable for answering fundamental biological questions about sympatric speciation, range expansion, and adaptive potential. A complete mitogenome is an informative reference for future studies on understanding the history of this species complex and future genetic studies of hybridization.

Total genomic DNA was extracted from a single gonadal tissue sample of *E.* sp. EZ collected from the Dhabiya reef site in the Persian/Arabian Gulf (24°21′55.8″N 54°06′02.9″E) with the DNeasy Blood and Tissue Kit (QIAGEN ). High-quality DNA was submitted for PCR-free library preparation and whole genome sequencing on an Illumina HiSeq3000 (100 bp paired-end reads) and a NextSeq500 (100 bp paired-end reads) at the University of Florida Interdisciplinary Center for Biotechnology Research. Trimmomatic v0.38 (Bolger et al. 2014) was used for adapter removal and quality filtering. Platanus v1.2.1 (Kajitani et al. 2014) was used to assemble reads into contigs and scaffolds with default parameters and K-mer ranging from 49 to 99. From the first pass genome assembly, we successfully identified a complete mitochondrial chromosome using the published *E. mathaei* mitogenome sequence (accession number: NC_034767.1). Annotations of the mitogenome were computed using Geneious v11.1.4 (Kearse et al. 2012) with the published *E. mathaei* mitogenome sequence as a reference. Given the complex phylogenetics of this species complex, we used BLASTn searches at the National Center for Biotechnology Information (NCBI) with cytochrome c oxidase subunit I gene (COI) to confirm that our sample matched *E.* sp. EZ. Phylogenetic analysis of eight *Echinometra* COI sequences (derived from six *Echinometra* species), suggests that the ‘*E. mathaei*’ mitogenome previously deposited at NCBI would more accurately be defined taxonomically as *E.* sp. EZ (Sequence data and maximum likelihood tree available from the Dryad Digital Repository: https://doi.org/10.5061/dryad.20k3fj0).

**Figure 1:**
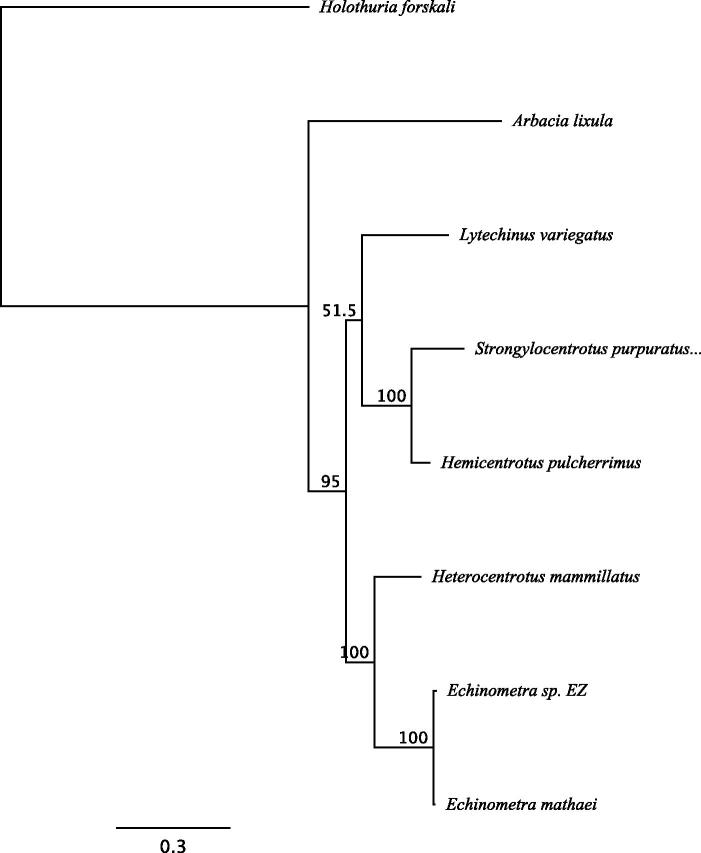
The maximum likelihood (ML) tree generated using Geneious v11.1.4 with six Echinoidea species: *Strongylocentrotus purpuratus* (accession number: X12631.1), *Lytechinus variegatus* (NC_037785.1), *Heterocentrotus mammillatus* (NC_034768.1), *Hemicentrotus pulcherrimus* (NC_023771.1), *Echinometra mathaei* (NC_034767.1), *Arbacia lixula* (NC_001770.1), and one Holothuroidea as an outgroup: *Holothuria forskali* (FN562582.1). The ML tree was generated with an alignment of the whole mitogenome sequences of all species, using the GTR + G model. The numbers above the branches specify bootstrap percentages (1000 replicates).

The mitogenome of *E*. sp. EZ is 15,698 bp in length and contains 13 protein-coding genes, 22 tRNAs, 2 rRNAs, and a non-coding region with an identical organization to other Echinoidea (accession number: MH685644). The mitogenome generated here was aligned to the published *E. mathaei* genome using the MAFFT alignment with the E-INS-i algorithm implemented in Geneious v11.1.4. We found 147 SNPs differentiating the two individuals (∼99.1% identity) (SNP table available on the Dryad Digital Repository: https://doi.org/10.5061/dryad.20k3fj0). Using this same alignment protocol, we generated a 16,520 column nucleotide alignment of the mitogenomes from *Echinometra* sp. EZ, *Echinometra mathaei*, *Arbacia lixula*, *Lytechinus variegatus*, *Strongylocentrotus purpuratus*, *Hemicentrotus pulcherrimus*, *Heterocentrotus mammillatus*, and the outgroup *Holothuria forskali* (Holothuroidea) (Alignment data are available on the Dryad Digital Repository: https://doi.org/10.5061/dryad.20k3fj0). With this alignment, we used MEGA v7.0.21 (Kumar et al. 2016) to determine that the best fit model of nucleotide substitution was GTR + G. We then generated a maximum likelihood (ML) tree using this model in the PhyML v3.0 plugin (Guindon et al. 2010) in Geneious v11.1.4 (with 1000 bootstrap replicates). The ML tree is in agreement with other published phylogenies based on morphological characteristics and mitochondrial regions (Figure 1) (Smith et al. 2006; Kroh and Smith 2010).

## References

[CIT0001] Arakaki Y, Uehara T 1999 Morphological comparison of black Echinometra individuals among those in the Indo-West Pacific. Zoolog Sci. 16:551–558.

[CIT0002] Bolger AM, Lohse M, Usadel B 2014 Trimmomatic: a flexible trimmer for Illumina sequence data. Bioinformatics. 30:2114–2120. 24695404 10.1093/bioinformatics/btu170 PMC4103590

[CIT0003] Bronstein O, Loya Y 2013 The taxonomy and phylogeny of Echinometra (Camarodonta: Echinometridae) from the Red Sea and Western Indian Ocean. PLoS ONE. 8:e77374 24116225 10.1371/journal.pone.0077374 PMC3792913

[CIT0004] Guindon S, Dufayard J-F, Lefort V, Anisimova M, Hordijk W, Gascuel O 2010 New algorithms and methods to estimate maximum-likelihood phylogenies: assessing the performance of PhyML 3.0. Syst Biol. 59:307–321. 20525638 10.1093/sysbio/syq010

[CIT0005] Kajitani R, Toshimoto K, Noguchi H, Toyoda A, Ogura Y, Okuno M, Yabana M, Harada M, Nagayasu E, Maruyama H, et al. 2014 Efficient de novo assembly of highly heterozygous genomes from whole-genome shotgun short reads. Genome Res. 24:1384–1395. 24755901 10.1101/gr.170720.113 PMC4120091

[CIT0006] Kearse M, Moir R, Wilson A, Stones-Havas S, Cheung M, Sturrock S, Buxton S, Cooper A, Markowitz S, Duran C, et al. 2012 Geneious Basic: an integrated and extendable desktop software platform for the organization and analysis of sequence data. Bioinformatics. 28:1647–1649. 22543367 10.1093/bioinformatics/bts199 PMC3371832

[CIT0007] Kroh A, Smith AB 2010 The phylogeny and classification of post-palaeozoic echinoids. J Syst Palaeontol. 8:147–212.

[CIT0008] Kumar S, Stecher G, Tamura K 2016 MEGA7: Molecular evolutionary genetics analysis version 7.0 for bigger datasets. Mol Biol Evol. 33:1870–1874. 27004904 10.1093/molbev/msw054 PMC8210823

[CIT0009] Lessios H 2006. Speciation in sea urchins. Echinoderms. Durham Proceedings of the 12th International Echinoderm Conference 91–101.

[CIT0010] McCartney MA, Keller G, Lessios HA 2000 Dispersal barriers in tropical oceans and speciation in Atlantic and eastern Pacific sea urchins of the genus Echinometra. Mol. Ecol. 9:1391–1400. 10972777 10.1046/j.1365-294x.2000.01022.x

[CIT0011] Mita M, Uehara T, Nakamura M 2004 Speciation in four closely related species of sea urchins (genus Echinometra) with special reference to the acrosome reaction. Invertebr Repr Dev. 45:169–174.

[CIT0012] Moulin L, Grosjean P, Leblud J, Batigny A, Collard M, Dubois P 2015 Long-term mesocosms study of the effects of ocean acidification on growth and physiology of the sea urchin Echinometra mathaei. Marine Environ Res. 103:103–114. 10.1016/j.marenvres.2014.11.009 25490159

[CIT0013] Palumbi SR, Grabowsky G, Duda T, Geyer L, Tachino N 1997 Speciation and population genetic structure in tropical Pacific sea urchins. Evolution. 51:1506–1517. 28568622 10.1111/j.1558-5646.1997.tb01474.x

[CIT0014] Smith AB, Pisani D, Mackenzie-Dodds JA, Stockley B, Webster BL, Littlewood TJ 2006 Testing the molecular clock: molecular and paleontological estimates of divergence times in the Echinoidea (Echinodermata). Mol Biol Evol. 23:1832–1851. 16777927 10.1093/molbev/msl039

